# Strategies to Generate Endorsement on Campus: Aligning AFU Principles With University Strategic Plans and Goals

**DOI:** 10.1093/geroni/igaa057.1749

**Published:** 2020-12-16

**Authors:** Cassandra Barragan, Sarah Walsh, Andrea Zakrajsek

**Affiliations:** Eastern Michigan University, Ypsilanti, Michigan, United States

## Abstract

Over the course of two semesters, Aging Studies Program faculty affiliates at Eastern Michigan University generated interest and support in the pursuit of applying for the AFU designation. During this time, we learned that when the AFU principles were aligned with university strategic plans and goals of various academic units, we were overwhelmingly endorsed at all levels of the university. By using the AFU principles as foundation for our efforts, we approached stakeholders with a value-added perspective. This approach ensured that when we did receive endorsement, we also maintained the integrity of the AFU principles while also speaking to administrative and departmental concerns, such as enrollment, intergenerational programs, and community engagement. We will discuss strategies to build interest at all levels of the university when pursuing or building your AFU designation and provide ways to identify relevant stakeholders to build capacity while seeking endorsement for joining the AFU global network.

